# Associations between 24-hour movement compositions and cardiometabolic health in children and adolescents: a five-part compositional analysis using data from the International Children’s Accelerometery Database (ICAD)

**DOI:** 10.1136/bmjsem-2025-002568

**Published:** 2025-06-12

**Authors:** Zoë A Marshall, Adam Runacres, Pedro Curi Hallal, Russ Jago, Soyang Kwon, Kate Northstone, Russell R Pate, Jardena Puder, John J Reilly, Luis B Sardinha, Niels Wedderkopp, Ester Van Slujis, On behalf of the International Children’s Accelerometery Database (ICAD) Collaborators, LB Andersen

**Affiliations:** 1 Institute of Sport, Manchester Metropolitan University, Manchester, UK; 2 Intelligent Health, Reading, UK; 3 University of Illinois at Urbana-Champaign, Urbana, Illinois, USA; 4 Population Health Sciences, Bristol Medical School, University of Bristol, Bristol, UK; 5 Buehler Center for Health Policy and Economics, Northwestern University Feinberg School of Medicine, Chicago, Illinois, USA; 6 Department of Exercise Science, University of South Carolina, Columbia, South Carolina, USA; 7 Obstetric Service, Lausanne University Hospital Department of Woman Mother and Child, Lausanne, Switzerland; 8 Department of Psychological Sciences and Health, University of Strathclyde, Glasgow, UK; 9 Exercise and Health Laboratory, CIPER, Faculdade de Motricidade Humana, Universidade de Lisboa, Lisbon, Portugal; 10 Department of Clinical Research, University of Southern Denmark, Odense, Denmark; 11 MRC Epidemiology Unit, University of Cambridge, Cambridge, UK

**Keywords:** Physical activity, Accelerometer, Children, Adolescent

## Abstract

**Objectives:**

The benefits of physical activity (PA) and the negative impacts of sedentary time (SED) on health in youth are well established. However, uncertainty remains surrounding how PA and SED jointly influence cardiometabolic risk (CMR) factors. The aim of this study was to determine the joint influence of SED, light-, moderate- and vigorous-intensity PA (LPA, MPA and VPA), and sleep on CMR factors using five-part compositional analyses.

**Methods:**

Data were pooled from 16 cohort studies comprising 22 474 children and adolescents from the International Children’s Accelerometery Database. PA was measured using hip-mounted accelerometers with sleep self-reported. CMRs included body mass index (BMI), waist circumference (WC), systolic and diastolic blood pressure, fasting high- and low-density lipoprotein cholesterol, triglycerides, insulin and glucose. Time spent for sleep, SED, LPA, MPA and VPA was analysed using a compositional linear regression model.

**Results:**

The overall PA composition explained between 3.0 and 27.0% of the variance in CMR factors after accounting for age, sex, ethnicity and seasonal variation. However, when movement behaviours were explored in isolation, only sleep was associated with all CMR factors. In girls, compositions with 10 min more VPA were associated with a 2.5–4.4% greater BMI and WC. However, 10 min reallocations of time in boys had no impact on any CMR factor.

**Conclusion:**

These findings highlight that sleep and VPA are significantly associated with all CMR factors in youth, and therefore specific recommendations are needed to improve the current, and future, health of children and adolescents.

**WHAT IS ALREADY KNOWN ON THIS TOPIC**

- Physical activity (PA) in children and adolescents is strongly associated with cardiometabolic health.
- Different movement behaviours when studied in isolation have significant, and independent, effects on cardiometabolic health in children and adolescents.

**WHAT THIS STUDY ADDS**

- This study furthers the understanding of the independent, and interactive, effect of movement behaviours on cardiometabolic health in children and adolescents.
- Sleep and vigorous intensity PA were highlighted as key indicators for favourable cardiometabolic health.
- 10 min reallocation of PA in boys, irrespective of intensity, had no significant impact on any cardiometabolic risk factor.

**HOW THIS STUDY MIGHT AFFECT RESEARCH, PRACTICE OR POLICY**

- Future PA guidelines should highlight the importance of higher-intensity PA and meeting sleep recommendations to promote favourable cardiometabolic health in children and adolescents.

## Introduction

 The relationship between physical activity (PA) and cardiometabolic health is well documented in a variety of population groups.^1^,^5^ Markers for cardiometabolic risk (CMR), such as atherosclerosis and cardiovascular disease (CVD), begin in early childhood, with factors such as obesity, sedentariness and physical inactivity suggested to increase the progression of risk throughout childhood.^1^ PA in paediatric populations has been shown to be a protective factor for current, and future CMR^6^; therefore, the risk of developing CVD and metabolic conditions later in life may be significantly reduced for those meeting PA recommendations.

The majority of our understanding of the relationship between PA and health outcomes still revolves around moderate to vigorous PA (MVPA), with a developing understanding of the influence of remaining behaviours (ie, sedentary time (SED), sleep and light-intensity PA (LPA)) that make up on average 95% of a child’s day.^5^ The intensity of PA is recognised as important for cardiometabolic health, as demonstrated by the WHO PA guidelines setting explicit targets for daily MVPA in children and adolescents.^7^ However, the collapsing of moderate and vigorous movements into one metric, MVPA, may be masking the importance of higher-intensity movement.^8^,^10^ Indeed, previous research exploring the impact of higher-intensity movement suggests that moderate-intensity movement may not be a sufficient stimulus to ‘trigger potential cardiometabolic benefits in healthy children’.^10^ More specifically, a number of studies highlight the importance of VPA in children and adolescents, with those who engage in higher volumes of VPA shown to have more favourable outcomes in adiposity,^11 12^ cardiovascular health^10 13^ and cardiorespiratory fitness.^8 14^ Higher-intensity movement is linked to higher fitness and lower adiposity, but on the other end of the intensity spectrum, evidence suggests that time spent on LPA may also be beneficial for markers of cardiovascular health, primarily systolic and diastolic blood pressures.^15^ Studies using compositional analyses and isotemporal substitution, which models the predicted outcomes in health markers when time is reallocated from one behaviour to another, indicate that engaging in PA, regardless of intensity, is beneficial for cardiovascular health.^16 17^ These results are in conflict with earlier studies suggesting that total SED accrued is independently associated with CMR than PA alone.^18 19^

While the relationship between PA and cardiometabolic health is well established, a greater understanding is required of their interaction with SED and sleep given that the majority of any 24-hour period is spent in one of these behaviours.^20^ As such, an integrated approach is necessary to better understand how health outcomes relate to the 24-hour composition of movement behaviours. This integrated approach has been applied previously by a number of authors, including advocates for compositional analysis including Dumuid *et al*^21^ and Chastin *et al*.^22^ The use of compositional approaches controls for the inherently codependent nature of movement behaviours to explore the collective and independent associations of time spent in each intensity of movement with health outcomes.^21 22^ As a result, compositional analysis can overcome issues of collinearity often found with traditional approaches^20^,^22^ and can also model how reallocating time from one behaviour to another could impact health outcomes.^23^ Research that employed compositional analysis in children and adolescents found the combined associations of time in waking movement to be important for cardiometabolic health. More specifically, compositions with more time in VPA were particularly favourable for markers of adiposity, body mass index (BMI) z-score (BMI-z), waist circumference (WC) and high-density lipoprotein cholesterol (HDL-C) levels,^11^ while days with short sleep and high SED were linked to increased adiposity and risk of metabolic syndrome in children.^24^ Furthermore, Carson *et al*^20^ found MVPA and sleep were negatively associated with CMR markers, BMI-z and WC, and blood pressure, with reallocation of time away from MVPA to remaining behaviours unfavourable for BMI-z. The associations observed by Carson *et al*^20^ were supported somewhat by more recent evidence suggesting more time spent in MVPA relative to remaining waking behaviours was favourable for adiposity using fat-free mass.^25^

The application of compositional analysis in the context of movement behaviours and health has primarily focused on the use of four-part analyses; therefore, these studies have collapsed MPA and VPA into MVPA^20 25^ or if exploring the intensity of PA have focused on waking behaviours, omitting sleep.^11^ Some studies have applied a five-part analysis to explore the impact of the full spectrum of movement behaviours on V̇O_2max_ in children and adolescents^8^ and the effect of differing lengths of sedentary bouts in adults^26^,^28^; however, to the best of the author’s knowledge, no studies have assessed CMR in youth using a five-part compositional analysis approach. Subsequently, the aim of this study was to examine the independent, and interactive, effects of the five-movement behaviours (sleep, SED, LPA, MPA and VPA) on CMR factors in children and adolescents. The second aim was to model how changes in behaviour at all intensities may influence cardiometabolic markers through isotemporal substitutions.

## Materials and methods

### Participants

Participants were from the International Children’s Accelerometery Database (ICAD; http://www.mrc-epid.cam.ac.uk/research/studies/icad/) which is a harmonised database containing objectively measured PA and SED from children and adolescents from 21 population studies across 10 countries.^2 29^ All sleep data contained within ICAD is self-reported. The aims, study design criteria, participant inclusion criteria and harmonisation process have been previously described elsewhere.^29^ All raw data within ICAD were collected between 1998 and 2009. Participants of the 16 studies which provided accelerometer-measured PA and SED, and self-reported sleep data in combination with at least one of the cardiometabolic outcome measures of interest constituted the sample for the present cross-sectional analysis (n=22 474). The 16 cohort studies included in the present study were conducted across 8 countries (Brazil, Denmark, Estonia, Norway, Portugal, Switzerland, the United Kingdom and the United States) and included children and adolescents between the ages of 6 and 18 years. This study complies with the Declaration of Helsinki; all study protocols were approved by the institutional ethics committees where the study was conducted; and informed assent was obtained from all participants and their parent/guardian where appropriate.

### Equity, diversity and inclusion statement

The author group is gender balanced and consists of junior, mid-career and senior researchers located all over the globe. Our study population included both male and female adolescents from different socioeconomic backgrounds across eight countries. While we covary for all demographic factors within our analyses, our study population had >85% white participants and thus the findings might not be generalisable to other ethnically or culturally diverse groups of children and adolescents.

### Patient and public involvement

Patients and/or the public were not involved in the design, conduct, reporting or dissemination plans of this research.

### Data analyses

After the processing of the PA data and cardiometabolic health outcomes (described in detail in the online supplemental material: *Assessment of Physical Activity and Cardiometabolic Health Outcomes*), each day was expressed as a five-part movement composition encompassing time spent in SED, LPA, MPA, VPA and sleep. Linear predictive models were used to predict changes in cardiometabolic health outcomes, with sex-specific smallest worthwhile changes (SWC) calculated for each parameter using the formula: 0.2*group SD^30^ (online supplemental table 1).

All compositional analyses were conducted in R (http://cran.r-project.org) using the compositions package (V.1.40-2) and all relevant dependencies.^22^ A detailed description of the compositional analysis methodology can be found in the online supplemental material (see Compositional Data Analysis). All traditional statistical analyses were conducted in SPSS V.26 (IBM, Portsmouth, UK). Sex differences in anthropometric characteristics and cardiometabolic health outcomes were assessed using a one-way ANCOVA. Cohen’s *d* was also calculated, with ≤0.20, ≥0.21 to ≤0.60, ≥0.61 to ≤0.80 and ≥0.81 considered a trivial, moderate, large and very large effect, respectively.^31^ Prior to publication, we reviewed our statistical analysis and presentation for consistency with the CHecklist for statistical Assessment of Medical Papers (CHAMP) statement.^32^ The full CHAMP checklist is available in the online supplemental material.

## Results

Of the original 22 474 participants initially extracted from the ICAD, 12 536 were excluded for failing to meet the wear-time criteria, or having missing cardiometabolic health data, leaving 9938 (5325 girls; 11.4±1.8 years) children and adolescents with valid accelerometery data. Participants within the final sample provided an average of 5.8±1.3 valid days of accelerometer data with an average wear time of 13.2±1.7 hours∙day^−1^. The number of participants with the corresponding cardiometabolic health measures varied, and a full breakdown of the number of participants with complete accelerometery and cardiometabolic health markers is provided in table 1.

**Table 1:** Participant descriptive statistics

	Boys (n=4613)	Girls (n=5325)
Descriptives		
Age (years)	11.4±1.7	11.4±1.8
Height (cm)	147.9±12.0	147.6±10.8
Weight (kg)	41.1±11.9	41.6±11.4*
Ethnicity	85.1% White	84.6% White
	0.3% Black	0.3% Black
	0.8% Asian	0.8% Asian
	13.8% Not reported	14.3% Not reported
Cardiometabolic health outcomes (n, %)		
BMI (kg∙m^2^)	18.4±3.1(n=4613, 100.0%)	18.8±3.4†(n=5325, 100.0%)
BMI z-score	−0.06±1.0(n=4613, 100.0%)	0.05±1.0†(n=5325, 100.0%)
Fasting glucose (mmol∙l^−1^)	5.1±0.4(n=1193, 25.9%)	5.0±0.4†(n=1436, 27.0%)
Insulin (pmol∙l^−1^)	48.7±29.7(n=1190, 25.8%)	57.5±35.5†(n=1430, 26.9%)
HDL-C (mmol∙l^−1^)	1.5±0.3(n=2736, 59.3%)	1.4±0.3†(n=3097, 58.2%)
LDL-C (mmol∙l^−1^)	2.3±0.6(n=2734, 59.3%)	2.5±0.6†(n=3093, 58.1%)
Fasting triglycerides (mmol∙l^−1^)	0.9±0.5(n=2722, 59.0%)	1.0±0.5†(n=3071, 57.7%)
Systolic blood pressure (mmHg∙min^−1^)	105±14(n=3736, 81.0%)	105±10*(n=4235, 79.5%)
Diastolic blood pressure (mmHg∙min^−1^)	59±7(n=3736, 81.0%)	60±7†(n=4235, 79.5%)
Waist circumference (cm)	66.2±9.2(n=4606, 99.8%)	64.9±8.9†(n=5319, 99.9%)

All values are presented as mean±SD unless otherwise stated. All statistics for cardiometabolic outcomes were covaried for age and ethnicity.

* Indicates a significant difference between boys and girls (p<0.05).

† Indicates a significant difference between boys and girls (p<0.01).

BMI, body mass index; HDL-C, high-density lipoprotein cholesterol; LDL-C, low-density lipoprotein cholesterol.

There were no differences in any anthropometric measurements between boys and girls which were included or excluded (p>0.05). Additionally, there was no sex difference for any anthropometric parameter (p>0.05) except weight where girls were heavier than their male peers (p<0.05; table 1). After covarying for age and ethnicity, girls had a higher BMI (+0.4 kg∙m^2^, p<0.01, d=0.12 (0.08–0.16)), BMI z-score (+0.01, p<0.01, d=0.11 (0.07–0.15)), insulin (+8.8 pmol∙l^−1^, p<0.01, d=0.12 (0.08–0.16)), LDL-C (+0.2 mmol∙l^−1^, p<0.01, d=0.33 (0.28–0.39)), triglycerides (+0.1 mmol∙l^−1^, p<0.01, d=0.20 (0.15–0.25)) and diastolic blood pressure (+1 mmHg∙min^−1^, d=0.14 (0.10–0.19); table 1) than boys. However, boys had higher fasting glucose (+0.1 mmol∙l^−1^, p<0.01, d=0.25 (0.17–0.33)), HDL-C (+0.1 mmol∙l^−1^, p<0.01, d=0.33 (0.28–0.39)), WC (+1.2 cm, p<0.01, d=0.14 (0.10–0.18)) and systolic blood pressure (p<0.05, d=0.00 (−0.04 to 0.04); table 1).

### PA composition description

In boys, the geometric means highlight that the largest portion of the day was spent in sleep (48.1%), followed by LPA (24.4%), with VPA only accounting for 1.0% of the day (table 2). Similarly, girls spent the longest portion of the day sleeping (47.3%), but this was followed by SED (25.4%), with VPA accounting for just 0.8% of the day. Boys spent significantly less time on sedentary (F_(1,9,860)_ = 36.5, p<0.01, d=−0.19 (−0.15 to −0.23)) and significantly more time on MPA (F_(1,9,860)_ = 1341.8, p<0.01, d=0.70 (0.66–0.74)), VPA (F_(1,9,860)_ = 687.5, p<0.01, d=1.32 (1.28–1.37)) and sleep F_(1,9,860)_ = 108.4, p<0.01, d=0.06 (0.02–0.10)) than girls. However, there was no significant difference between boys and girls for LPA (F_(1,9,860)_ = 1.2, p=0.27, d=0.02 (−0.02 to 0.06)). LPA and MPA and LPA and SED demonstrated the smallest pairwise variation, indicating high codependency, whereas VPA had the largest pairwise log-ratio variation when compared with all other movement behaviours indicating less codependency (table 3). The ILR models revealed that the overall PA composition significantly predicted all cardiometabolic health outcomes explaining between 3.2% and 23.0% of the variance after covarying for age, sex, ethnicity and PA seasonal variation (table 4). When individual movement behaviours were considered, after adjusting for covariates and the other movement behaviours, only sleep was significantly associated with all cardiometabolic health outcomes (table 4). Contrastingly, SED, LPA, MPA and VPA were associated with some, but not all, cardiometabolic health outcomes. Data displaying the 95% CIs for all ILR model behaviours are available in online supplemental table 2.

**Table 2:** Time spent on physical activity, sedentary time and sleep for boys and girls expressed as the unadjusted overall mean, geometric mean and a percentage of 24 hours

	Overall mean (min∙day^−1^)	Geometric mean (min∙day^−1^)	% of 24 hours
Boys			
SED	311.9	342.1	23.8
LPA	311.5	350.7	24.4
MPA	39.5	40.5	2.8
VPA	16.8	14.0	1.0
Sleep	594.8	692.7	48.1
Girls			
SED	325.4*	365.8*	25.4*
LPA	309.4	348.5	24.2
MPA	28.1*	30.2*	2.1*
VPA	10.6*	11.5*	0.8*
Sleep	604.5*	681.1*	47.3*

* Indicates a significant difference between boys and girls (p<0.01).

LPA, light physical activity; MPA, moderate physical activity; SED, sedentary time; VPA, vigorous physical activity.

**Table 3:** Pairwise log ratio variation matrix in the full sample (n=9938)

	SED	LPA	MPA	VPA	Sleep
SED		0.05	−0.10	−0.22	0.09
LPA	0.05		−0.03	−0.15	0.05
MPA	−0.10	−0.03		0.07	−0.07
VPA	−0.22	−0.15	0.07		−0.18
Sleep	0.09	0.05	−0.07	−0.18	

LPA, light physical activity; MPA, moderate physical activity; SED, sedentary time; VPA, vigorous physical activity.

**Table 4:** Compositional ILR behaviour models for the cardiometabolic health parameters

	Model p value	Model R^2^	Y_SED_	p	Y_LPA_	p	Y_MPA_	p	Y_VPA_	P	Y_Sleep_	P
Log BMI (kg∙m^2^)	<0.001	0.122	0.004	<0.001	0.004	0.010	0.001	0.167	−0.005	<0.001	−0.004	0.003
BMI z-score	<0.001	0.126	0.067	<0.001	0.037	0.060	0.029	0.020	−0.080	<0.001	−0.052	0.003
Log fasting glucose (mmol∙l^−1^)	<0.001	0.174	0.003	0.044	0.004	0.004	−0.002	0.002	0.001	0.339	0.004	0.002
Log insulin (pmol∙l^−1^)	<0.001	0.230	0.015	0.006	0.015	0.059	−0.012	0.008	−0.003	0.318	−0.016	0.043
Log HDL-C (mmol∙l^−1^)	<0.001	0.045	−0.002	0.312	−0.004	0.990	0.001	0.927	0.002	0.067	0.005	0.041
Log LDL-C (mmol∙l^−1^)	<0.001	0.050	−0.009	<0.001	−0.005	0.093	−0.002	0.210	−0.003	0.018	0.019	<0.001
Log fasting triglycerides (mmol∙l^−1^)	<0.001	0.068	0.028	<0.001	0.023	<0.001	−0.002	0.637	0.001	0.610	−0.050	<0.001
Systolic blood pressure (mmHg∙min^−1^)	<0.001	0.071	0.611	<0.001	0.443	0.051	−0.417	0.004	−0.104	0.190	−0.532	<0.001
Diastolic blood pressure (mmHg∙min^−1^)	<0.001	0.032	0.055	0.601	−0.330	0.034	−0.332	<0.001	−0.054	0.321	0.660	<0.001
Log waist circumference (cm)	<0.001	0.178	0.007	<0.001	0.003	0.004	0.003	<0.001	−0.004	<0.001	−0.009	<0.001

All models were covaried for age, sex, ethnicity and month of physical activity monitoring.

BMI, body mass index; HDL-C, high-density lipoprotein cholesterol; ILR, isometric log ratios; LDL-C, low-density lipoprotein cholesterol; LPA, light physical activity; MPA, moderate physical activity; SED, sedentary time; VPA, vigorous physical activity.

### Association between PA composition and cardiometabolic health outcomes

Change matrices describing the effect of reallocating 10 min of time from one behaviour to another in boys and girls are presented in table 5. In boys, there was no effect of displacing 10 min of any movement behaviour on any of the 10 cardiometabolic health parameters measured with all of the predicted changes smaller than the SWC. Contrastingly, in girls’ compositions with 10 min less VPA (<1.5 min∙day^−1^) compared with the average 11.5 min∙day^−1^ were associated with a 4.17–4.32% higher BMI and a 2.50–2.76% larger WC. Moreover, in girls with compositions <1.5 min∙day^−1^ of VPA were increased with a higher BMI-z score 0.25–0.26 units higher than the average (0.05), and compositions with <21.5 min∙day^−1^ were associated with a decreased BMI-z score between 0.05 and 0.07 units (table 5).

**Table 5:** Change matrices of reallocating 10 min of time from the behaviour in columns to the behaviour in rows on cardiometabolic health outcomes in boys and girls, expressed as a percentage change around the sample mean

Boys						Girls					
	SED	LPA	MPA	VPA	Sleep		SED	LPA	MPA	VPA	Sleep
**BMI (kg∙m** ^**2**^ **)**											
SED		0.24	0.12	1.96	0.24	SED		0.22	0.08	4.39*	0.23
LPA	−0.24		−0.11	1.72	0.00	LPA	−0.23		−0.14	4.17*	0.00
MPA	−0.14	0.10		1.82	0.10	MPA	−0.11	0.12		4.28*	0.12
VPA	−1.20	−0.96	−1.07		−0.95	VPA	−1.60	−1.37	−1.51		−1.37
Sleep	−0.24	0.02	−0.11	1.72		Sleep	−0.23	0.00	−0.14	4.17*	
**BMI z-score**											
SED		−16.25	−8.47	−134.48	−16.30	SED		62.02	22.39	1169.47*	61.93
LPA	16.54		7.69	−118.32	−0.14	LPA	−63.04		−39.27	1107.81*	0.27
MPA	9.79	−6.84		−125.07	−6.89	MPA	−31.24	32.15		1139.60*	32.06
VPA	82.18	65.56	73.33		65.51	VPA	−435.86*	−372.47*	−412.10*		−372.56*
Sleep	16.63	0.00	7.78	−118.23		Sleep	−63.12	0.27	−39.36	1107.72*	
**Fasting glucose (mmol∙l** ^**−1**^ **)**											
SED		−0.06	0.41	0.08	−0.16	SED		−0.06	0.65	0.45	−0.16
LPA	0.06		0.47	0.15	−0.10	LPA	0.06		0.71	0.51	−0.10
MPA	−0.30	−0.36		−0.22	−0.46	MPA	−0.43	−0.49		0.02	−0.59
VPA	−0.01	−0.07	0.40		−0.17	VPA	−0.05	−0.12	0.59		−0.22
Sleep	0.16	0.10	0.57	0.25		Sleep	0.16	0.10	0.81	0.61	
**Insulin (pmol∙l** ^**−1**^ **)**											
SED		1.31	2.46	1.62	0.87	SED		1.08	2.68	2.77	0.71
LPA	−1.32		1.16	0.32	−0.43	LPA	−1.09		1.61	1.70	−0.36
MPA	−2.11	−0.77		−0.47	−1.21	MPA	−2.09	−0.99		0.69	−1.37
VPA	−1.27	0.07	1.21		−0.38	VPA	−1.23	−0.13	1.47		−0.50
Sleep	−0.89	0.44	1.59	0.75		Sleep	−0.72	0.38	1.91	2.06	
**HDL-C (mmol∙l** ^**−1**^ **)**											
SED		−0.10	−0.30	−0.19	−0.10	SED		−0.10	−0.41	−0.36	−0.10
LPA	0.11		−0.20	−0.09	0.00	LPA	0.10		−0.31	−0.26	0.00
MPA	0.26	0.15		0.06	0.16	MPA	0.32	0.22		−0.04	0.22
VPA	0.15	0.05	−0.15		0.05	VPA	0.18	0.08	−0.23		0.08
Sleep	0.10	0.00	−0.20	−0.09		Sleep	0.10	0.00	−0.31	−0.26	
**LDL-C (mmol∙l** ^**−1**^ **)**											
SED		−0.21	0.16	0.86	−0.40	SED		−0.19	0.32	2.25	−0.36
LPA	0.22		0.37	1.07	−0.19	LPA	0.19		0.50	2.43	−0.17
MPA	−0.07	−0.29		0.78	−0.47	MPA	−0.17	−0.36		2.07	−0.53
VPA	−0.41	−0.62	−0.26		−0.81	VPA	−0.67	−0.87	−0.36		−1.04
Sleep	0.40	0.18	0.55	1.25		Sleep	0.36	0.17	0.67	2.60	
**Fasting triglycerides (mmol∙l** ^**−1**^ **)**											
SED		0.12	0.65	−0.79	0.73	SED		0.10	0.73	−2.35	0.68
LPA	−0.14		0.52	−0.92	0.60	LPA	−0.12		0.62	−2.46	0.57
MPA	−0.61	−0.47		−1.38	0.13	MPA	−0.64	−0.52		−2.98	0.02
VPA	0.30	0.43	0.96		1.04	VPA	0.61	0.72	1.35		1.30
Sleep	−0.74	−0.60	−0.07	−1.51		Sleep	0.68	−0.57	−2.35	3.02	
**Systolic blood pressure (mmHg∙min** ^**−1**^ **)**											
SED		0.11	0.49	0.21	0.11	SED		0.11	0.49	0.21	0.11
LPA	−0.11		0.38	0.10	0.00	LPA	−0.11		0.38	0.10	0.00
MPA	−0.38	−0.27		−0.17	−0.27	MPA	−0.39	−0.27		−0.17	−0.27
VPA	−0.13	−0.02	0.36		−0.02	VPA	−0.14	−0.02	0.36		−0.02
Sleep	−0.11	0.00	0.38	0.10		Sleep	0.11	0.00	0.38	0.10	
**Diastolic blood pressure (mmHg∙min** ^**−1**^ **)**											
SED		0.10	0.36	0.21	0.01	SED		0.10	0.49	0.43	0.01
LPA	−0.10		0.25	0.10	−0.09	LPA	−0.10		0.39	0.33	−0.09
MPA	−0.29	−0.19		−0.09	−0.28	MPA	−0.37	−0.27		0.06	−0.36
VPA	−0.14	−0.04	0.22		−0.13	VPA	−0.18	−0.08	0.31		−0.17
Sleep	−0.01	0.09	0.10	0.19		Sleep	−0.01	0.09	0.48	0.42	
**Waist circumference (cm)**											
SED		0.21	0.05	1.27	0.27	SED		0.21	−0.03	2.76*	0.27
LPA	−0.22		−0.16	1.05	0.06	LPA	−0.22		−0.24	2.55*	0.06
MPA	−0.09	0.13		1.19	0.19	MPA	−0.04	0.18		2.73*	0.23
VPA	−0.81	−0.59	−0.75		−0.53	VPA	−1.08	−0.86	−1.10		−0.81
Sleep	−0.27	−0.05	−0.22	1.00		Sleep	−0.27	−0.05	−0.29	2.50*	

* Indicates a change above the smallest worthwhile change (%).

BMI, body mass index; HDL-C, high-density lipoprotein cholesterol; LDL-C, low-density lipoprotein cholesterol; LPA, light physical activity; MPA, moderate physical activity; SED, sedentary time; VPA, vigorous physical activity.

## Discussion

This study examined the inter-related effects of all movement behaviours (SED, LPA, MPA, VPA and sleep) using a five-part compositional analysis approach on cardiometabolic health in a large sample of children and adolescents. The main findings of the present study were that the overall movement composition was significantly associated with all cardiometabolic health outcomes explaining between 3.2 and 27.0% of the variance after covarying for age, sex, ethnicity and seasonal variation in PA. Moreover, sleep was the only movement behaviour which was significantly independently associated with all CMR factors. The results also suggest compositions with 10 min increases in VPA, irrespective of the behaviour it replaces, predict a 2.7–4.6% reduction in WC and BMI, and reduce BMI-z by 0.1 units in girls. However, a 10 min reallocation of VPA had a nonsignificant impact on all CMR factors in boys, highlighting that the intensity of PA may be of paramount importance in determining CMR in girls.

It is widely accepted that PA provides health benefits for children and adolescents with the benefits of being physically active thought to have a protective effect on adulthood.^33^ The results of this study support the proposed protective effect as the overall PA composition explained between 3.2 and 27.0% of the variance in CMR factors after covarying for age, sex, ethnicity and PA seasonal variation. This is in agreement with Carson *et al*^11^ who found that for children, the overall PA composition was significantly associated with WC, BMI, SBP, DBP, HDL-C and glucose. However, in discord with the current study, Carson *et al*^11^ reported that the overall PA composition was not significantly associated with LDL-C cholesterol, triglycerides or insulin. The disparity in results could be explained, at least in part, by Carson *et al*^11^ additionally covarying for socioeconomic status, smoking, daily sodium intake, saturated fat and energy all of which significantly impact cardiometabolic health.^34^ However, this is unlikely to fully explain the differences between these studies with other studies reporting conflicting results for other health outcomes including WC,^20^ arterial stiffness and pulse wave velocity,^35 36^ and mental health outcomes.^37 38^ Consequently, the findings thus far from compositional analysis studies in children and adolescents seem equivocal, as a recent systematic review highlighted in adults^39^ and young childhood.^40^ This could be due to methodological differences in both PA data collection and the different techniques used to quantify health parameters.^39 40^ Therefore, future research with consistent methodology around PA measurement and analysis process is needed, so the health impacts of 24-hour compositions can be fully elucidated.

When time was allocated to VPA, the isotemporal substitution indicated that it would be associated with a decreased WC, BMI and BMI-z in girls only which was surprising. However, this is not the first time that VPA in isolation has demonstrated a greater association with cardiometabolic health outcomes than MVPA in children.^3 9 10 14^ More specifically, Nyström *et al*^10^ reported stronger associations between VPA and CVD risk factors than MVPA both cross-sectionally and longitudinally aligning with the findings of a meta-analysis exploring the association between VPA and health-related outcomes.^9^ The result of the current study tentatively supports the notion that VPA is more strongly related to cardiometabolic health outcomes than other movement behaviours and furthers it by revealing that sex differences may be evident for the first time. One possible explanation for the sex difference is that the association with VPA could be due to the sex differences in VPA undertaken in the present study (boys: 16.8±14.4 min∙day^−1^; girls: 10.6±8.8 min∙day^−1^); however, this is similar to other large-scale population-based studies in this similar age group.^41^ More specifically, a 10 min reallocation of VPA equates to a 92.6% change in VPA volume for girls compared with a 59.5% VPA volume change in boys, which might influence the magnitude of change predicted even though the same amount of time has been reallocated. Considering the limited success of PA interventions to date^42 43^ and the relatively small changes reported even when interventions are successful, the current findings indicate that a drastic change to our approach to PA in youth may be needed.

In the current study, the average self-reported sleep duration was 594.8±52.2 min∙day^−1^ (9.9 hours∙night^−1^) for boys and 604.5±53.3 min∙day^−1^ (10.0 hours∙night^−1^) for girls which are both within the recommended sleep guidelines for children aged 6–12 years.^44^ Interestingly, sleep was the only movement behaviour in isolation that significantly predicted all cardiometabolic health outcomes after accounting for age, sex, ethnicity or PA seasonal variation. In adults, a ‘U’-shaped relationship between sleep duration and cardiovascular outcomes occurs with both short (≤6 hours∙night^−1^) and long durations (≥8 hours∙night^−1^) posing an increased risk of CVD.^45 46^ However, the relationship between sleep and cardiometabolic health in children and adolescents is less well understood with evidence suggesting that short sleep duration is associated with an increase in some, but not all, CMR factors.^47^ More specifically, short sleep duration has been strongly linked to obesity,^48^ increased blood pressure^49^ and a reduced basal metabolic rate^50^ due to alterations in autonomic, metabolic and endothelial functions which influence both hormone levels and eating behaviours in children and adolescents.^51^ However, the evidence on abnormal sleep patterns and dyslipidaemia or irregular resting glucose/insulin remains inconsistent, and there is a need for more high-quality evidence using objective measures of sleep duration with methodological inconsistencies likely contributing to the equivocal findings.^47^

### Research/policy implications

While lifestyle factors such as a healthy diet and the promotion of regular PA have been routinely cited in international policies and guidelines to reduce the incidence of cardiovascular and cardiometabolic diseases, sleep has often been overlooked,^24^ until recently. More specifically, sleep is now being recognised as one of the pillars for a healthy lifestyle,^52^ and 24-hour PA guidelines, including specific sleep recommendations, have now been implemented in countries in North America,^53^ Europe^54^ and Australasia.^55 56^ However, all of the 24-hour movement guidelines presently still advocate for increasing MVPA, whereas the current study indicates that MPA and VPA should not be combined into a single metric given their distinct effects on CMR factors. Collapsing MPA and VPA into MVPA potentially masks the importance of PA intensity for health and supports the notion that children and adolescents, particularly girls, may require a vigorous stimulus to realise health benefits.^8 12 37^ While some of the effect sizes in this study, such as the associations with HDL-C and systolic blood pressure, are modest, others like insulin and fasting glucose show stronger associations. These findings suggest that VPA may have a more pronounced impact on cardiometabolic outcomes, particularly for specific populations such as girls. Thus, even small effect sizes, when applied across large populations, can have meaningful public health implications. Taken together, this evidence indicates future studies should report the effects of MPA and VPA separately or report the MPA/VPA ratio of MVPA so the influence of PA on health can be explored in more detail. Moreover, there is growing evidence suggesting the need to promote VPA among youth for optimal health, and the guidelines for MVPA might need to be revised to incorporate specific VPA guidelines.

### Limitations

While there are strengths to the current study including a sample size of ~10 000 children and adolescents with objectively measured PA and the use of a five-part compositional analysis, there are limitations which must be acknowledged. First, all accelerometery data within this study were collected using hip-worn accelerometers, so movements involving limited vertical hip movement (ie, cycling) or water-based activities (where the accelerometers have to be removed) are likely to be misclassified. Additionally, sleep was collected using self-report measures, and although self-report sleep closely matches polysomnography,^57^ further research using objective measures is needed to confirm the findings in the present study. Moreover, the domain of PA from the accelerometery data could not be determined as well as several other confounding factors including diet, pubertal and socioeconomic status potentially inflating the influence of PA, and these should be controlled for in future studies. Additionally, the current study also had 85% white participants and even though ethnicity was covaried in all analyses, the results of this study should be interpreted in the context of explaining the association of PA and CMR factors in white children and adolescents.

## Conclusion

The overall five-part PA composition was a significant predictor of all cardiometabolic health outcomes independent of age, sex, ethnicity and seasonal variation. However, sleep was the only movement behaviour that was significantly associated with all cardiometabolic health outcomes, even when the proportion of time spent on other behaviours was considered. Moreover, reallocating time to VPA had distinct effects by sex, emphasising the need for future studies to report the individual levels of MPA and VPA or report the MPA/VPA ratio to ascertain the relative importance of PA intensity for the current, and future, health of children and adolescents.

## Supplementary material

10.1136/bmjsem-2025-002568 online supplemental material 1

## Data Availability

Data may be obtained from a third party and are not publicly available.
