# Supplementary material for: Associations between 24-hour movement compositions and cardiometabolic health in children and adolescents: a five-part compositional analysis using data from the International Children’s Accelerometery Database (ICAD)
Source: BMJ Open Sport Exerc Med. 2025 Jun 12;11(2):e002568. doi: 10.1136/bmjsem-2025-002568 (PMC12164611; doi:10.1136/bmjsem-2025-002568)
Supplement: online supplemental material 1 [file bmjsem-11-2-s001.docx]

**The associations between 24-hour movement compositions and cardiometabolic health in children and adolescents: A five-part compositional analysis using data from the International Childrens Accelerometery Database (ICAD)**

Marshall, Z.A. ^1,2^, Runacres, A. ^1^*, Hallal, P.C. ^3^, Jago, R. ^4^, Kwon, S. ^5^, Northstone, K. ^4^, Pate, R. ^6^, Puder, J.J. ^7^, Reilly, J.J. ^8^, Sardinha, L.B. ^9^, Wedderkopp, N. ^10^, van Sluijs, E.M.F. ^11^, On behalf of the International Children’s Accelerometery Database (ICAD) Collaborators^^^

**Supplementary Material**

Contents

[Methods 2](#_Toc198046433)

[Assessment of Physical Activity and Cardiometabolic Health Outcomes 2](#_Toc198046434)

[Compositional Data Analysis 3](#_Toc198046435)

[CHAMP: CHecklist for statistical Assessment of Medical Papers 4](#_Toc198046436)

[Results 5](#_Toc198046437)

[Supplementary Material Table 1: Smallest worthwhile change calculations for all cardiometabolic health outcomes 5](#_Toc198046438)

[Supplementary Table 2: Compositional ILR behaviour models, with 95% confidence intervals for the cardiometabolic health parameters 6](#_Toc198046439)

[References 7](#_Toc198046440)

# Methods

## Assessment of Physical Activity and Cardiometabolic Health Outcomes

An overview of the assessment of physical activity, sedentary time, and sleep will be described here; however, a full description is available elsewhere (1). Briefly, all raw accelerometer waist-worn data from all longitudinal studies included within the ICAD were reprocessed and reanalysed to harmonise all of the physical activity, sedentary time, and sleep variables across studies using specialist software (KineSoft version 3.3.20, Saskatchewan, Canada; <http://www.kinesoft.org>). All accelerometer files were re-integrated into 60 s epochs with non-wear time periods were defined as ≥ 60 minutes of consecutive zeroes, allowing for 2 minutes of non-zero interruptions. The inclusion criteria for accelerometery data within the current study was a valid wear time of ≥ 10 hours∙day^-1^ on any 3 days similar to previous studies of this type (2, 3), to ensure a valid representation of habitual PA was gathered. Time spent sedentary, in LPA, MPA, and VPA were determined by applying the Evenson cut points (4). Sleep was ascertained from subjective estimates of sleep duration, as participants were instructed to remove the accelerometer whilst sleeping, which have been described in detail elsewhere (5). Briefly, four studies collected information about the usual times children went to- and got out of bed (6-9) and two studies asked when children usually went to sleep and woke up (10, 11). Despite the differing methods of Sleep estimation, it has been demonstrated that both parent- and child-reported sleep closely matches polysomnography, the gold standard for measuring Sleep assessment (12). Therefore, all data was harmonised within ICAD to provide estimations of Sleep duration in children and adolescents.

Participant characteristics including age, sex, and ethnicity were recorded for all studies and used for descriptive purposes. Anthropometric measures of height (m) and weight (kg) were measured by trained personnel in standardised formats across all studies from this BMI and BMI Z-scores were calculated. Five cardiometabolic variables (glucose [GLU], insulin [INS], high density lipoprotein cholesterol [HDL-C], low density lipoprotein cholesterol [LDL-C] and triglycerides [TRI]) across 8 studies (6, 7, 10, 13-16) assessed fasting lipid and glucose metabolism using a venous blood sample. Resting systolic (SBP) and diastolic (DBP) blood pressure were measured in 12 studies (6, 7, 10, 11, 13-18). All blood pressure measurements were conducted with participants in a rested condition and reported as an average measure of two, three, or four recordings. Waist circumference (WC) was measured in all 16 studies (6-11, 13-20) and is a good proxy measure of abdominal adiposity (21). Five out of the 16 studies (6, 17) reported WC as a mean of two measurements whereas the remaining 11 reported a singular WC measurement.

## Compositional Data Analysis

Firstly, geometric means were computed to denote the proportion of time spent in each PA behaviour, or Sleep, by expressing each movement behaviour after normalisation as a proportion of total time (22, 23). Variance matrices were then calculated to provide an insight into the co-dependency, and dispersion, between movement behaviours by measuring the variance between pair-wise log ratios (23, 24). More specifically, a ratio tending towards zero indicates high co-dependency between variables with numbers further from zero highlighting less co-dependency. To determine the relative effect of the overall PA composition on cardiometabolic health outcomes sequential linear models were created by rotating each of the five movement behaviours (SED, LPA, MPA, VPA, Sleep) via isometric log ratio transformations (ILR). The first coefficient and its p-value were reported for each rotation to determine the extent to which the individual movement behaviour was associated with the cardiometabolic health outcome variable, relative to the other movement behaviours (2, 3, 24, 25). Moreover, the overall model *p* value and R^2^ value were reported to ascertain the variance explained by the overall movement composition. Finally, isotemporal substitution modelling were performed to predict the change in each cardiometabolic health outcome by systematically re-allocating 10 minutes from one movement behaviour to another (3, 22, 23, 26). All predictive change matrices were expressed as a percentage change relative to the compositional mean, with significant changes identified as any change greater than the SWC (%).

## CHAMP: CHecklist for statistical Assessment of Medical Papers

| ***Design and Conduct*** | | | |
| --- | --- | --- | --- |
| Clear description of the goal of research, study objective(s), study design, and study population | Yes | Unclear | No |
| Clear description of outcomes, exposures/treatments and covariates, and their measurement methods | Yes | Unclear | No |
| Validity of study design | Yes | Unclear | No |
| Clear statement and justification of sample size | Yes | Unclear | No |
| Clear declaration of design violations and acceptability of the design violations | Yes | Unclear | No |
| Consistency between the paper and its previously published protocol | Yes | Unclear | No |
| ***Data Analysis*** | | | |
| Correct and complete description of statistical methods | Yes | Unclear | No |
| Valid statistical methods used and assumptions outlined | Yes | Unclear | No |
| Appropriate assessment of treatment effect or interaction between treatment and another covariate | Yes | Unclear | No |
| Correct use of correlation and associational statistical testing | Yes | Unclear | No |
| Appropriate handling of continuous predictors | Yes | Unclear | No |
| Confidence intervals do not include impossible values | Yes | Unclear | No |
| Appropriate comparison of baseline characteristics between the study arms in randomized trials | Yes | Unclear | No |
| Correct assessment and adjustment of confounding | Yes | Unclear | No |
| Avoiding model extrapolation not supported by data | Yes | Unclear | No |
| Adequate handling of missing data | Yes | Unclear | No |
| ***Reporting and Presentation*** | | | |
| Adequate and correct description of the data | Yes | Unclear | No |
| Descriptive results provided as occurrence measures with confidence intervals, and analytic results provided as association measures and confidence intervals along with P-values | Yes | Unclear | No |
| Confidence intervals provided for the contrast between groups rather than for each group | Yes | Unclear | No |
| Avoiding selective reporting of analyses and P-hacking | Yes | Unclear | No |
| Appropriate and consistent numerical precisions for effect sizes, test statistics, and P-values, and reporting the P-values rather their range | Yes | Unclear | No |
| Providing sufficient numerical results that could be included in a subsequent meta-analysis | Yes | Unclear | No |
| Acceptable presentation of the figures and tables | Yes | Unclear | No |
| ***Interpretation*** | | | |
| Interpreting the results based on association measures and 95% confidence intervals along with P-values, and correctly interpreting large P-values as indecisive results, not evidence of absence of an effect | Yes | Unclear | No |
| Using confidence intervals rather than post-hoc power analysis for interpreting the results of studies | Yes | Unclear | No |
| Correctly interpreting occurrence or association measures | Yes | Unclear | No |
| Distinguishing causation from association and correlation | Yes | Unclear | No |
| Results of pre-specified analyses are distinguished from the results of exploratory analyses in the interpretation | Yes | Unclear | No |
| Appropriate discussion of the study methodological limitations | Yes | Unclear | No |
| Drawing only conclusions supported by the statistical analysis and no generalization of the results to subjects outside the target population | Yes | Unclear | No |

# Results

## **Supplementary Material Table 1:** Smallest worthwhile change calculations for all cardiometabolic health outcomes

|  | Boys SD | Boys SWC | SWC (% of mean) | Girls SD | Girls SWC | SWC (% of mean) |
| --- | --- | --- | --- | --- | --- | --- |
| BMI (kg∙m^2^) | 3.1 | 0.6 | 3.3% | 3.4 | 0.7 | 3.7% |
| BMI Z-Score | 1.0 | 0.2 | 200% | 1.0 | 0.2 | 200% |
| Fasting Glucose (mmol∙l^-1^) | 0.4 | 0.08 | 1.6% | 0.4 | 0.08 | 1.6% |
| Insulin (pmol∙l^-1^) | 29.7 | 5.94 | 12.2% | 35.5 | 7.10 | 12.3% |
| HDL-C (mmol∙l^-1^) | 0.3 | 0.06 | 4.0% | 0.3 | 0.06 | 4.3% |
| LDL-C (mmol∙l^-1^) | 0.6 | 0.12 | 5.2% | 0.6 | 0.12 | 4.8% |
| Triglycerides (mmol∙l^-1^) | 0.5 | 0.10 | 11.1% | 0.5 | 0.10 | 10.0% |
| Systolic Blood Pressure (mmHg∙min^-1^) | 14.0 | 2.8 | 2.7% | 10.0 | 2.0 | 1.9% |
| Diastolic Blood Pressure (mmHg∙min^-1^) | 7.0 | 1.4 | 2.4% | 7.0 | 1.4 | 2.3% |
| Waist Circumference (cm) | 9.2 | 1.8 | 2.7% | 8.9 | 1.8 | 2.8% |

SD = Standard Deviation, SWC = Smallest Worthwhile Change, % = Percentage, BMI = Body Mass Index, HDL-C = High Density Lipoprotein, LDL-C = Low Density Lipoprotein.

## **Supplementary Table 2**: Compositional ILR behaviour models, with 95% confidence intervals for the cardiometabolic health parameters

|  | Y_SED_ | 95% CI  (Lower, Upper) | Y_LPA_ | 95% CI  (Lower, Upper) | Y_MPA_ | 95% CI  (Lower, Upper) | Y_VPA_ | 95% CI  (Lower, Upper) | Y_Sleep_ | 95% CI  (Lower, Upper) |
| --- | --- | --- | --- | --- | --- | --- | --- | --- | --- | --- |
| Log BMI (kg∙m^2^) | 0.004 | (0.002,0.006) | 0.004 | (0.001,0.008) | 0.001 | (-0.001,0.003) | -0.005 | (-0.003,0.001) | -0.004 | (-0.006,-0.002) |
| BMI Z-Score | 0.067 | (0.039,0.093) | 0.037 | (-0.002,0.076) | 0.029 | (0.005,0.053) | -0.080 | (-0.094,-0.066) | -0.052 | (-0.085,-0.019) |
| Log Fasting Glucose (mmol∙l^-1^) | 0.003 | (0.001,0.005) | 0.004 | (0.002,0.006) | -0.002 | (-0.004,0.001) | 0.001 | (-0.001,0.003) | 0.004 | (0.002,0.006) |
| Log Insulin (pmol∙l^-1^) | 0.015 | (0.003,0.027) | 0.015 | (-0.001,0.031) | -0.012 | (-0.022,-0.002) | -0.003 | (-0.009,0.003) | -0.016 | (-0.032,0.001) |
| Log HDL-C (mmol∙l^-1^) | -0.002 | (-0.006,0.002) | -0.004 | (-0.01,0.002) | 0.001 | (-0.005,0.003) | 0.002 | (0.001,0.004) | 0.005 | (0.001,0.009) |
| Log LDL-C (mmol∙l^-1^) | -0.009 | (-0.004,0.004) | -0.005 | (-0.011,0.001) | -0.002 | (-0.006,0.002) | -0.003 | (-0.005,-0.001) | 0.019 | (0.013,0.025) |
| Log Fasting Triglycerides (mmol∙l^-1^) | 0.028 | (0.020,0.036) | 0.023 | (0.013,0.033) | -0.002 | (-0.008,0.004) | 0.001 | (-0.003,0.005) | -0.050 | (-0.060,-0.040) |
| Systolic Blood Pressure (mmHg∙min^-1^) | 0.611 | (0.309,0.913) | 0.443 | (-0.002,0.888) | -0.417 | (-0.699,-0.135) | -0.104 | (-0.261,0.053) | -0.532 | (-0.924,-0.140) |
| Diastolic Blood Pressure (mmHg∙min^-1^) | 0.055 | (-0.153,0.263) | -0.330 | (-0.636, -0.024) | -0.332 | (-0.526,-0.138) | -0.054 | (-0.160,0.052) | 0.660 | (0.397,0.923) |
| Log Waist Circumference (cm) | 0.007 | (0.006,0.008) | 0.003 | (0.001,0.005) | 0.003 | (0.002,0.004) | -0.004 | (-0.005,-0.003) | -0.009 | (-0.011,-0.007) |

All models were covaried for age, sex, ethnicity, and month of physical activity monitoring. Statistically significant associations (p < 0.05) are highlighted in bold. ILR = Isometric Log Ratios, SED = Sedentary Time, LPA = Light Physical Activity, MPA = Moderate Physical Activity, VPA = Vigorous Physical Activity, BMI = Body Mass Index, HDL-C = High Density Lipoprotein, LDL-C = Low Density Lipoprotein.

# References

1. Sherar LB, Griew P, Esliger DW*, et al.* International children's accelerometry database (ICAD): Design and methods. BMC Public Health. 2011;11(1):485.

2. Carson V, Tremblay M, Chaput J*, et al.* Compositional analyses of the associations between sedentary time, different intensities of physical activity, and cardiometaolic biomarkers among children and youth from the United States. PLoS One. 2019;14(7):e0220009.

3. Runacres A, MacKintosh KA, Chastin S*, et al.* The associations of physical activity, sedentary time, and sleep with V˙O2max in trained and untrained children and adolescents: A novel five-part compositional analysis. PLoS One. 2023;18(3):e0275557.

4. Evenson KR, Catellier DJ, Gill KI*, et al.* Calibration of two objective measures of physical activity for children. Journal of Sport Sciences. 2008;26(14):1557-65.

5. Collings PJ, Grøntved A, Jago R*, et al.* Cross-sectional and prospective associations of sleep duration and bedtimes with adiposity and obesity risk in 15 810 youth from 11 international cohorts. Pediatr Obes. 2022;17(4):e12873.

6. Riddoch C, Edwards D, Page A*, et al.* The European Youth Heart Study—Cardiovascular Disease Risk Factors in Children: Rationale, Aims, Study Design, and Validation of Methods. Journal of Physical Activity and Health. 2005;2(1):115-29.

7. Zahner L, Puder JJ, Roth R*, et al.* A school-based physical activity program to improve health and fitness in children aged 6-13 years ("Kinder-Sportstudie KISS"): study design of a randomized controlled trial [ISRCTN15360785]. BMC Public Health. 2006;6(1471-2458 (Electronic)):147.

8. Page AS, Cooper AR, Griew P*, et al.* Independent mobility, perceptions of the built environment and children's participation in play, active travel and structured exercise and sport: the PEACH Project. International Journal of Behavioral Nutrition and Physical Activity. 2010;7.

9. Niederer I, Kriemler S, Zahner L*, et al.* Influence of a lifestyle intervention in preschool children on physiological and psychological parameters (Ballabeina): study design of a cluster randomized controlled trial. BMC Public Health. 2009;9(1):94.

10. Golding J, Pembrey MF, Jones R. ALSPAC-the Avon Longitudinal Study of Parents and Children. I. Study methodology. 15. 2001:7-17.

11. van Sluijs EMF, Skidmore PML, Mwanza K*, et al.* Physical activity and dietary behaviour in a population-based sample of British 10-year old children: the SPEEDY study (Sport, Physical activity and Eating behaviour: environmental Determinants in Young people). BMC Public Health. 2008;8:388.

12. Combs D, Goodwin JL, Quan SF*, et al.* Mother Knows Best? Comparing Child Report and Parent Report of Sleep Parameters With Polysomnography. J Clin Sleep Med. 2019;15(1):111-7.

13. Hasselstrøm HA, Karlsson MK, Hansen SE*, et al.* A 3-Year Physical Activity Intervention Program Increases the Gain in Bone Mineral and Bone Width in Prepubertal Girls but not Boys: The Prospective Copenhagen School Child Interventions Study (CoSCIS). Calcif Tissue Int. 2008;83(4):243-50.

14. Centers for Disease Control and Prevention. Laboratory Procedures Manual. Atlanta; 2005.

15. Troiano RP, Berrigan D., Dodd KW*, et al.* Physical activity in the United States measured by accelerometer. Med Sci Sports Exerc. 2008;40:181-8.

16. Boyd A, Golding J, Macleod J*, et al.* Cohort Profile: the 'children of the 90s'--the index offspring of the Avon Longitudinal Study of Parents and Children. Int J Epidemiol. 2013;42(1):111-27.

17. Reilly JJ, Kelly L, Montgomery C*, et al.* Physical activity to prevent obesity in young children: cluster randomised controlled trial. Br Med J. 2006;333:1041.

18. Victora CG, Hallal P.C., Araújo CL*, et al.* Cohort profile: the 1993 Pelotas (Brazil) birth cohort study. Int J Epidemiol. 2008;37:704-9.

19. Levy SM, Warren JJ, Davis CS*, et al.* Patterns of fluoride intake from birth to 36 months. J Public Health Dent. 2001;61(0022-4006 (Print)):70-7.

20. Stevens J, Murray DM, Catellier DJ*, et al.* Design of the Trial of Activity in Adolescent Girls (TAAG). Contemp Clin Trials. 2005;26(2):223-33.

21. Ross R, Neeland IJ, Yamashita S*, et al.* Waist circumference as a vital sign in clinical practice: a Consensus Statement from the IAS and ICCR Working Group on Visceral Obesity. Nature Reviews Endocrinology. 2020;16(3):177-89.

22. Chastin S, Palarea-Albaladejo J, Dontje M*, et al.* Combined Effects of Time Spent in Physical Activity, Sedentary Behaviors and Sleep on Obesity and Cardio-Metabolic Health Markers: A Novel Compositional Data Analysis Approach. PLoS One. 2015;10(10).

23. Dumuid D, Stanford T, Martin-Fernández J*, et al.* Compositional data analysis for physical activity, sedentary time and sleep research. Stat Methods Med Res. 2018;27(12).

24. Dumuid D, Stanford T, Pedisic Z*, et al.* Adiposity and the isotemporal substitution of physical activity, sedentary time and sleep among school-aged children: a compositional data analysis approach. BMC Public Health. 2018;18(1).

25. Carson V, Tremblay M, Chaput J*, et al.* Associations Between Sleep Duration, Sedentary Time, Physical Activity, and Health Indicators Among Canadian Children and Youth Using Compositional Analyses. Appl Physiol Nutr Metab. 2016;41(6).

26. Janssen I, Clarke A, Carson V*, et al.* A systematic review of compositional data analysis studies examining associations between sleep, sedentary behaviour, and physical activity with health outcomes in adults. Applied physiology, nutrition, and metabolism = Physiologie appliquee, nutrition et metabolisme. 2020;45(10 (Suppl. 2)).
